# Integrin–fibronectin interaction is a pivotal biological and clinical determinant in papillary thyroid carcinoma

**DOI:** 10.1530/ERC-25-0101

**Published:** 2025-06-05

**Authors:** Domenico Rocco, Anna Tortora, Vincenzo Marotta, Aline Maria Machado, Heloisa S Selistre-de-Araújo, Mario Vitale

**Affiliations:** ^1^ Department of Medicine, Surgery and Dentistry, University of Salerno, Salerno, Italy; ^2^ UOC Clinica Endocrinologica e Diabetologica, AOU San Giovanni e Ruggi d’Aragona, Salerno, Italy; ^3^ Department of Physiological Sciences, Federal University of São Carlos, São Carlos, Brazil

**Keywords:** thyroid cancer, fibronectin, integrins, disintegrins

## Abstract

Integrins influence tumor growth, metastasis, and angiogenesis, making them potential targets for therapeutic intervention. In this study, we analyzed the TCGA mRNA-seq dataset to assess the expression levels of fibronectin (FN1) and associated integrin subunits, evaluating their relationship with clinical features in papillary thyroid cancer (PTC). These findings were further validated in a cell model. FN1 mRNA levels in BRAFV600E-positive PTC were 80-fold compared to normal thyroid tissue (NT), whereas PTC with RAS mutations exhibited FN1 levels similar to NT. ITGAV, encoding the αv integrin subunit, which pairs with β3 to form a receptor for FN, was also overexpressed in PTC. Elevated FN1 expression, and to a lesser extent ITGAV, correlated positively with lymph node metastasis, advanced cancer stages, extrathyroidal extension, and poorer prognoses. Patients in the highest quartile of FN1 expression had an increased risk of disease recurrence (OR = 7.277, 95% CI: 2.019–26.191, *P* < 0.0024). A non-tumoral thyroid cell line and two PTC cell lines were used as models to validate the mRNA-seq results. The proliferation and migration of a FN1 knock-out PTC cell mutant were significantly reduced and proliferation was restored upon the addition of soluble FN. DisBa-01, a recombinant RGD-disintegrin derived from *Bothrops alternatus* snake venom, which acts as an antagonist to the FN/αvβ3 interaction, inhibited PTC cell proliferation and migration. These results demonstrate that FN expression is a hallmark of aggressiveness in PTC. FN/αvβ3 interaction plays a pivotal role in PTC, suggesting that the FN/αvβ3 signaling is a potential therapeutic target for disintegrins or other molecules with similar action.

## Introduction

The extracellular matrix (ECM) of the basement membrane is a key regulator of numerous cellular processes in epithelial cells through intracellular signals engaging integrins and other cellular receptors (Miranti & Brugge 2002). Integrins are heterodimeric transmembrane receptors composed of 18 α-subunits and 8 β-subunits, which combine to form 24 distinct heterodimers in mammals. (Hynes 2002). These receptors are found in every cell where they mediate cell–ECM and cell–cell interactions, influencing adhesion, migration, differentiation, and survival. They bind ECM proteins, such as collagen (CG), laminin (LM), fibronectin (FN), vitronectin, and many others. The role of FN in tumorigenesis and malignant progression is controversial. Numerous reports demonstrate that FN in various types of tumor cells is associated with malignancy, metastasis, or poor prognosis (Han & Roman 2006, Mitra *et al.* 2011, Hicks *et al.* 2023). However, in some cancers, FN expression can act as a tumor suppressor inhibiting proliferation and dissemination (Taylor *et al.* 1998, Barney *et al.* 2020).

Normal thyroid cells have a limited integrin repertoire, with significant expression restricted to α3β1, α2β1, and αvβ3 (Vitale *et al.* 1993, Vitale *et al.* 1995, Hoffmann *et al.* 2005). Through these integrins, thyroid cells bind to three major ECM components: CG (via α2β1), LM (via α2β1 and α3β1), and FN (via αvβ3) (Takada *et al.* 2007). Increased expression of α2, α3, α4, α5, α6, and β4 integrin subunits has been documented in various thyroid tumor histotypes (Vitale *et al.* 1994, Serini *et al.* 1996, Dahlman *et al.* 1998, Hoffmann *et al.* 2005, Chernaya *et al.* 2018). The α3β1 integrin is overexpressed in papillary thyroid carcinoma (PTC), where it is involved in cell motility and invasion and is associated with advanced disease and poorer outcomes (Mautone *et al.* 2021). In normal thyrocytes, αvβ3/FN binding generates complex pleiotropic signaling, including the p21Ras/MAPK pathway and the PI-3K/Ca2+/calmodulin-dependent kinase II pathway (Illario *et al.* 2003*b*, Illario *et al.* 2005, Maddalena *et al.* 2005). These signals stimulate DNA synthesis, proliferation, and survival in both normal thyrocytes and PTC cells (Vitale *et al.* 1997, Vitale *et al.* 1998, Illario *et al.* 2003*a*, Maddalena *et al.* 2005, Rusciano *et al.* 2010). FN, assessed by immunohistochemistry, has been reported to be overexpressed in PTC compared to normal thyroid and adenoma and is significantly associated with malignancy (Prasad *et al.* 2005).

Genetic analysis identifies PTC subtypes (e.g., BRAF + TERT mutations) with aggressive behavior and worse prognosis, but the molecular mechanisms underlying the expansion and dissemination of this cancer are still unclear (Nikiforov & Nikiforova 2011). The advent of targeted tyrosine kinase inhibitors has improved the prognosis of aggressive thyroid carcinomas. However, treatment with BRAF inhibitors paradoxically can enhance FN expression, promoting a more invasive phenotype in BRAF-mutated thyroid cancer cells (Hicks *et al.* 2023). The aim of this study is to explore the role of integrins and FN in PTC, with a particular focus on the impact of driver gene mutations. The analysis of gene expression from the TCGA dataset, along with experimental evidence, demonstrates the pivotal role of the integrin/FN interaction in the biological and clinical behavior of PTC, identifying a novel therapeutic strategy.

## Materials and methods

### RNA-seq analysis

TCGA-THCA dataset was downloaded from the Cancer Genome Atlas (TCGA) (https://portal.gdc.cancer.gov/). We analyzed the raw-counts data with gene expression profile of 358 classical PTC (PTCcl); 37 tall cell PTC (PTCtc), and 58 solid tissue (NT) samples. Matching clinicopathological data were downloaded from cBioPortal for Cancer Genomics (https://www.cbioportal.org/) (Gao *et al.* 2013). Differential expression was reported as fold change ≥|1.5| along with associated adjusted *P* values (FDR ≤ 0.05), computed according to Benjamini–Hochberg (Benjamini & Cohen 2017). The quantification of RNA-seq data was obtained by the tool RNA-seq by expectation-maximization (RSEM) (Li & Dewey 2011).

### Cell lines and culture mediums

BCPAP (Leibnitz-Institut, Germany) and K1 (Merck KGaA, Germany) are PTC cell lines harboring BRAFV600E. K1 were cultured in DMEN/F12 (Euroclone S.p.A, Italy), 10% FBS (Gibco; Thermo Fisher Scientific, Inc., USA). Nthy-ori-3-1 is a human thyroid follicular cell line derived from normal thyroid tissue, immortalized by transfection with a plasmid containing a defective genome of the SV40 virus (SV-ori), allowing them to retain several characteristics of normal thyroid follicular cells. BCPAP and Nthy-ori-3-1 were cultured in RPMI-1640 (Euroclone), 10% FBS. Cells were cultured in a humidified incubator (5% CO_2_ and 95% air at 37°C).

### Western blot

The cells were lysed with lysis buffer (Tris HCl 50 mM pH 8, NaCl 120 mM, EDTA 5 mM, Triton 1%, NP40 1%, and protease inhibitors). To evaluate FN protein levels, 30 μg protein were electrophoresed on 7.5% SDS-PAGE and then transferred to nitrocellulose membranes, saturated with 5% non-fat dry milk in PBS/0.1% Tween 20. The membranes were then incubated overnight with rabbit monoclonal (MoAb) anti-FN antibody 1:1,000 (Abcam, UK) or mouse MoAb anti-β-tubulin 1:1,000 (Santa Cruz Biotechnology, USA). The following day, membranes were incubated for 1 h with peroxidase-conjugated anti-mouse or anti-rabbit IgG secondary antibody (Sigma-Aldrich, USA). Blots were developed using ECL (Elabscience) with ChemiDoc Imaging Systems (Bio Rad, USA).

### Flow cytometric analysis

MoAbs to integrin subunits were purchased from Santa Cruz Biotechnology; fluorescein-conjugated anti-mouse antibody was from Jackson Immunoresearch (UK). Cells were then analyzed by flow cytometry using a FACScan apparatus (Becton Dickinson Co., USA). Flow cytometric analysis was performed as follows: cells were harvested by trypsin-PBS, incubated with the primary MoAb for 1 h at room temperature (RT) in 0.5% BSA-PBS, washed with the same buffer, and incubated again with the secondary fluorescein-conjugated antibody for 30 min at RT. Cells were resuspended in BSA-PBS and analyzed by flow cytometry. Cytofluorimetric analysis of the cell cycle was performed as follows: cells were trypsinized, washed in PBS, and fixed in 70% cold ethanol for 30 min. After two PBS washes to remove ethanol, the cells were incubated overnight at 4°C in PBS containing 50 μg/mL propidium iodide, 10 μg/mL ribonuclease A, and DNase-free buffer. Flow cytometric analysis was then performed using a FACScan.

### Immunofluorescence

Cells were plated onto sterile glass coverslips and cultured at 37°C in culture medium, 10% FBS. Cells were fixed in 70% ethanol PBS for 10 min and blocked in 0.5% BSA for 10 min. Cells were incubated with mouse MoAb anti-human FN (Santa Cruz Biotechnology) in PBS and 0.2% Tween-20 for 1 h, washed in PBS, incubated with fluorescein-conjugated secondary antibody (Jackson Immunoresearch, USA) for 30 min, and observed with a fluorescence microscope (Carl Zeiss, Germany).

### Enzyme-linked immunoassay (ELISA)

A total of 10^4^ cells/well were plated in 96-well flat-bottomed microtiter plates (Costar, USA) in medium with 10% FBS and cultured. Then, cells were fixed by methanol–acetone (vol/vol) for 10 min at RT and air-dried. To measure FN in the culture supernatants, 5 μL culture medium was recovered from the wells, let dry in clean wells and fixed as above. Wells were filled with 100 μL MoAb antihuman FN in PBS, 0.5% BSA, and 0.2% Tween-20 and allowed to react for 1 h at 4°C. Then the plates were washed with PBS, filled with 100 μL horseradish peroxidase-conjugated mouse IgG (Sigma-Aldrich, USA) in PBS 0.2% Tween-20, allowed to react for 1 h, washed with PBS, and filled with 150 mL 1 mg/mL o-phenylenediamine, 0.006% hydrogen peroxide, and 0.1 mol/L citrate buffer, pH 5.0. After 30 min incubation, the absorbance at 450 nm was measured by a spectrophotometer.

### Cell spreading

The cells were plated in the presence of the RGD peptide (Arg-Gly-Asp) (Sigma-Aldrich), which inhibits FN/integrin binding, the ineffective control peptide RGE (Arg-Gly-Glu), or DiSba-01. After the indicated incubation time, the cells were examined by inverted phase contrast microscope at 3,400 magnification and photographed. For each experimental point, the surface of at least 50 cells was measured by the program ImageJ Version 1.54i (https://imagej.net/ij/), and the results were expressed as the mean ± SD.

### MTT assay

The cells were seeded in 96-well plates (1.5 × 10^4^ cells/well) coated with collagen and laminin. Cells were treated with different concentrations of human plasma fibronectin (Sigma-Aldrich) 0-1-5 μg/mL. After different time incubation, 4 mg/mL MTT (Sigma-Aldrich) was added to the cell medium and cells were cultured for a further 4 h in the incubator. The supernatant was removed, 100 μL/well DMSO were added, and the absorbance at 490 nm was measured. All experiments were performed in triplicate.

### Gelatin-Sepharose affinity chromatography

FN was depleted from the 10% FBS-containing DMEM/F12 medium by Gelatin-Sepharose affinity chromatography. Gelatin-Sepharose 4B (Cytiva, USA) was prepared according to the manufacturer’s instructions. The medium was passed through the Gelatin-Sepharose column, and the flow-through, devoid of FN, was collected. To confirm the successful depletion of FN, proteins from 10% FBS-containing medium, before and after Gelatin-Sepharose filtration, were precipitated using an acetone precipitation protocol. Briefly, an equal volume of ice-cold acetone was added to the medium samples, followed by overnight incubation at −20°C. The precipitated proteins were then collected by centrifugation at 12,000 *g* for 15 min at 4°C. The resulting protein pellets were washed with ice-cold 80% acetone, air-dried, resuspended, and analyzed by Western blotting.

### Migration assay

Cells were seeded at a density of 25,000 per 8 μm-pore insert in 24-well Transwell plates (Corning, USA) using serum-free DMEM/F12. The lower chambers were filled with DMEM/F12 supplemented with 5% FBS, from which fibronectin had been removed by Gelatin-Sepharose affinity chromatography. After 24 h of incubation at 37°C, non-migrated cells on the upper surface of the inserts were removed using cotton swabs. Migrated cells on the lower surface were fixed in ice-cold ethanol for 10 min and stained with 0.1% crystal violet at RT for 10 min. Excess stain was removed by rinsing with tap water. Images were acquired with an Olympus BX53 microscope at 200× magnification, capturing multiple fields per insert. Migrated cells were quantified using the ImageJ software. All experiments were conducted in triplicate.

### Generation of cell mutants

K1 cells (3 × 10^5^/well) were seeded in 6-well plates 24 h before transfection to achieve 50–70% confluence. To generate a FN1 knock-out, we applied the KN2.0 CRISPR system (Origene, USA). For transfection, 1 μg gRNA vector and 1 μg donor DNA were separately diluted in 250 μL Opti-MEM I. 4 μL TransIT-X2 (Mirus Bio, USA) reagent was added to the diluted DNA mixture and incubated for 20 min at RT. The mixture was then added dropwise to the cells, followed by gentle rocking of the plate. 48 h post-transfection, cells were split 1:10 and cultured for an additional 3 days. This process was repeated two more times. To select for puromycin-resistant cells, puromycin was added to the culture medium at a concentration determined by a kill curve (1 µg/mL). Media was changed every 2–3 days. Cloning rings were used to isolate individual cell colonies from low-density cultures (5% confluency) in 10 cm dishes. Puromycin selection was applied during seeding. Puromycin-resistant cells were analyzed for genomic editing by Western blotting (WB) using a fibronectin antibody to assess gene knockdown.

### DisBa-01 expression and purification

The expression and purification of DisBa-01 was performed as described (Ramos *et al.* 2008). Briefly, *E. coli* BL21(DE3) was transformed with plasmid pet28(a)DisBa-01. Protein expression was induced for 3 h, followed by lysis and purification in three steps: affinity chromatography (HIS-Select® HF Nickel Affinity Gel, Sigma-Aldrich; Code: P6611), size exclusion chromatography (Superdex 75 10/300 GL, GE Healthcare, Sweden; Code: 17-5174-01), and anion exchange chromatography (Mono-Q 5/50 GL, GE Healthcare; Code: 17-516601). Purified protein was dialyzed against water and frozen in aliquots.

### Statistical analysis

All data are presented as the mean ± standard deviation. Normal distribution was assessed by the Shapiro–Wilk statistic. Normally distributed variables were compared using the paired *T*-Student test and non-normally distributed variables by the Wilcoxon signed-ranks test. Comparisons of integrin expression and clinical features were conducted by one-way analysis of variance (ANOVA) and statistical significance was assumed for *P* < 0.01. Univariate regression analysis was performed using the Spearman rank correlation test. Data analyses were conducted with the SPSS Statistics version 26 (IBM Corp., USA).

### Confirmation of approval

Ethical approval from the local ethics committee was not required as no human material or data was used. The study was specifically approved by the institutional review board.

## Results

### mRNA expression of FN1 and integrin subunits binding FN in normal thyroid tissue and in PTC

**Figure 1:**
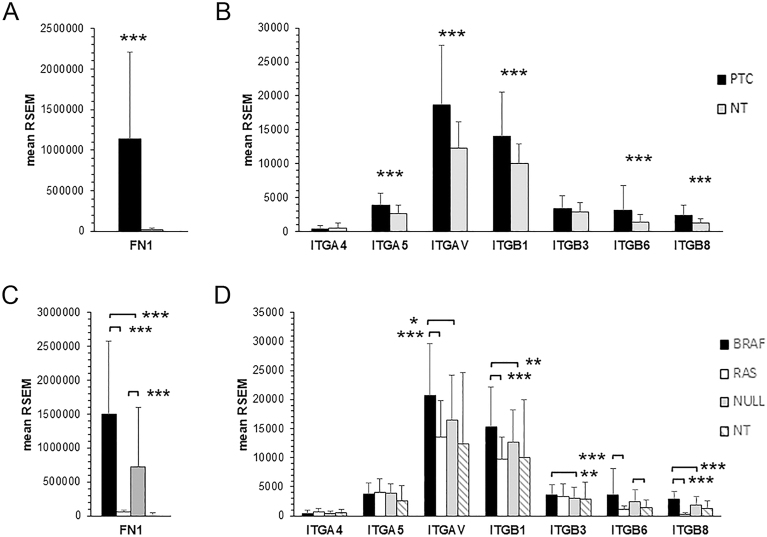
mRNA expression of FN1 (A) and integrin subunits binding FN (B) in PTC (black bars) and NT (gray bars). Correlation between FN1 (C) or integrin (D) expression and driver mutations in PTC. The gene expression was determined in PTC cases with BRAFV600E (BRAF, *n* = 204), RAS mutation (RAS, *n* = 19), or neither mutation (NULL, *n* = 132). Only PTCcl and PTCtc were considered. Data are presented as the mean ± standard deviation. Wilcoxon test: *P < 0.01, **P < 0.001, ***P < 0.00001; RNA-seq by expectation maximization.

We analyzed the RNA-seq of TCGA-THCA dataset. The results show the cumulative analysis of 355 PTC classic (PTCcl) and tall cell (PTCtc) variants and 58 normal thyroid tissues (NT) (Fig. 1). The follicular variant was excluded for the presence of NIFTP pathology subtype. FN1 mRNA in PTC was 60-fold more abundant than in NT (Fig. 1A). Integrin heterodimers binding FN are α4β1, α5β1, αVβ1, αVβ3, αVβ6, and αVβ8. The genes encoding the integrin subunits ITGAV, ITGB1, ITGB6, ITGB8 and to a lesser extent ITGA5 were all overexpressed in PTC (Fig. 1B). The analysis was further refined by examining the expression of FN1 and integrins in relation to driver gene mutations. Notably, BRAFV600E-positive PTC displayed the highest expression of FN1 and integrin subunits, whereas the expression profile of RAS-positive PTC was similar to that of NT (Fig. 1C). FN1 mRNA was 83-fold more abundant in PTC with BRAFV600E and 39-fold in PTC with neither BRAF nor RAS mutations compared to NT (both Wilcoxon *P* < 0.00001). In contrast, FN1 mRNA expression in PTC with RAS mutation was comparable with that of NT (55,051 ± 96,207 vs 18,195 ± 25,028, mean RSEM ± SD, Wilcoxon *P* = 0.177). ITGAV, ITGB1, ITGB6, and ITGB8 were confirmed to be overexpressed in BRAFV600E-positive PTC compared to NT (Fig. 1D). These genes were also overexpressed, albeit to a lesser extent, in PTC lacking both BRAF and RAS mutations. Notably, the expressions of all integrin subunits in PTC with RAS mutations were not significantly different from that of NT.

### Association of FN1, integrin expression and clinical features

**Figure 2:**
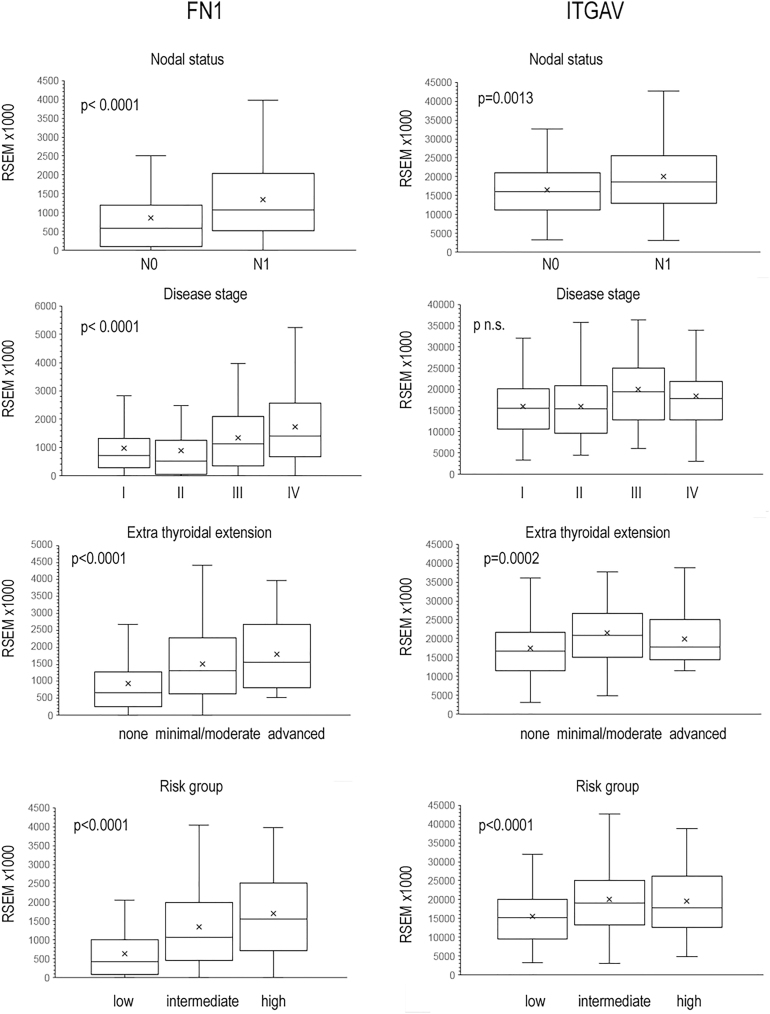
Correlation of FN1 and ITGAV mRNA expressions and clinical features of patients with PTC.

**Figure 3:**
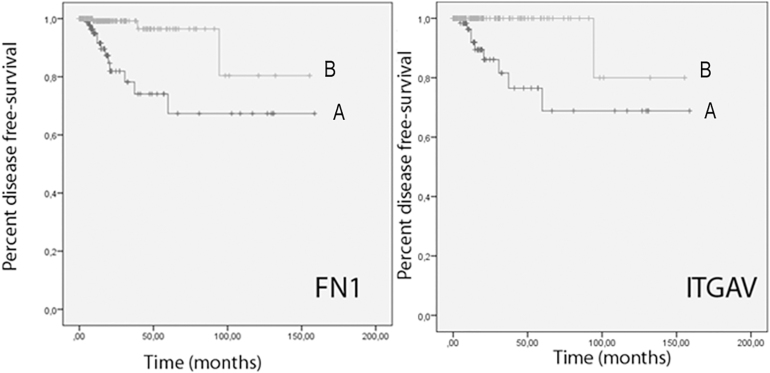
Kaplan–Meier analysis of disease-free survival of patients with FN1 and ITGAV mRNA expressions above (A) or below the median (B). FN1, *P* < 0.001; ITGAV, *P* = 0.002.

We analyzed the correlation of FN1, ITGA4, ITGA5, ITGAV, ITGB1, ITGB3 and ITGB6 with disease status, risk of tumor recurrence according to the 2015 American Thyroid Association guidelines (Haugen *et al.* 2015), and patient outcome. FN1 expression positively correlated with all parameters of advanced disease: lymph node metastasis, advanced stage, and extrathyroidal extension (for all *P* < 0.0001) (Table 1, Fig. 2). Higher ITGAV (Fig. 2) and ITGB1 expression were associated with lymph node metastasis and extrathyroidal extension, while ITGB6 expression positively correlated with lymph node metastasis, advanced stage, and extrathyroidal extension. In addition to FN1, high risk of tumor recurrence was associated with higher expression of ITGAV, ITGB1 and ITGB6. The TCGA dataset includes 287 patients with PTCcl or PTCtc who completed their follow-up period without disease recurrence (disease-free patients, DFP) and 23 patients who experienced disease recurrence (DRP). Among the 23 DRP, 18 had FN1 mRNA expression levels above the median (78.2%), with an odds ratio (OR) for recurrence of 4.110 (95% CI: 1.485–11.371), *P* < 0.0065 (Table 2). The OR for recurrence was even higher when quartiles were considered 7.277 (95% CI: 2.019–26.191), *P* < 0.0024. ITGAV expression also showed a positive correlation with worse outcomes. Seventeen DRP had ITGAV mRNA expression above the median (74%), with an OR for recurrence of 3.235 (95% CI: 1.239–8.442). The Kaplan–Meier analysis of disease-free survival revealed significantly worse outcomes for patients with FN1 mRNA expression above the median (log-rank *P* < 0.001) (Fig. 3A) and for those with ITGAV mRNA expression above the median (log-rank *P* = 0.0024) (Fig. 3B).

**Table 1:** Mean gene expression and analysis of the correlation with nodal status, disease stage, extrathyroidal extension and risk group*. Only significant data are reported.

Nodal status			
Gene	N0	N1	*P*
ITGAV	16,581	20,067	0.0013
ITGB1	12,546	14,923	0.0022
ITGB6	2,604	3,400	0.0007
ITGB8	2,056	2,570	0.0009
FN1	865,693	1,347,066	<0.0001

Disease stage					
	I	II	III	IV	*P*
ITGB6	2,716	2,158	4,220	3,156	0.0071
FN1	981,019	878,322	1,329,867	1,718,111	<0.0001

Extrathyroidal extension				
	None	Minimal	Moderate/advanced	*P*
ITGAV	17,409	21,593	19,851	0.0002
ITGB1	13,296	15,342	17,878	0.0025
ITGB6	2,735	3,779	4,516	0.0204
FN1	928,634	1,515,710	1,805,129	<0.0001

Risk group*				
	Low	Intermediate	High	*P*
ITGAV	15,595	20,138	19,590	<0.0001
ITGB1	12,213	14,654	16,393	0.0014
ITGB6	2,132	3,467	4,043	0.0035
FN1	626,613	1,342,289	1,693,896	<0.0001

* Estimated risk of tumor recurrence based on the 2015 American Thyroid Association guidelines. Statistical analysis was ANOVA for disease stage, extrathyroidal extension and risk group, and Wilcoxon for nodal status. The mean gene expression is calculated by RSEM.

**Table 2:** Disease outcome and high or low expression of FN1 and ITGAV with respect to the median.

	Number of patients		*OR*	*P*
	Low	High		
**FN1**				
DFP	153	134		<0.0065
DRP	5	18	4.110 (1.485–11.371)	
**ITGAV**				
DFP	153	134		0.0164
DRP	6	17	3.235 (1.239–8.442)	

DFP, disease-free patients; DRP, patients with disease recurrence; OR, odds ratio; *P*, chi square statistics.

### Integrin expression and FN production of thyroid cell lines

**Figure 4:**
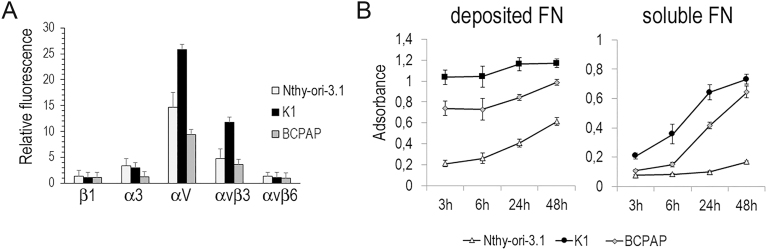
Integrin expression and FN production in Nthy-ori-3.1, BCPAP and K1 cells. (A) Integrin expression was determined by flow cytometry with specific antibodies. (B) Cells were cultured for the indicated time in 96-wells plates, the amount of immobilized FN (right) and soluble FN in the supernatant (left) were determined by ELISA. Relative FN content per well is expressed as the mean absorbance ± SD of triplicate wells.

A non-tumoral thyroid cell line (Nthy-ori-3.1) and two PTC cell lines harboring the BRAFV600E mutation (BCPAP and K1) were used as models to study the role of integrin–FN interactions. Based on mRNA expression analysis from the TCGA dataset, we assessed the membrane expression of FN-binding integrins in the three cell lines using flow cytometry with specific antibodies (Fig. 4A). The K1 cell line exhibited higher levels of αv and αvβ3 integrins compared to the other cell lines. To quantify the FN produced by these cells, the three cell lines were cultured in medium containing 10% FBS, after which the medium was removed and the adherent cells and insoluble matrix were fixed. The amount of deposited FN was measured by ELISA. Nthy-ori-3.1 cells deposited significantly less FN than the PTC cells (Fig. 4B). In addition, soluble FN in the culture supernatant was more abundant in PTC cells than in Nthy-ori-3.1 cells.

### FN promotes cell migration and proliferation

**Figure 5:**
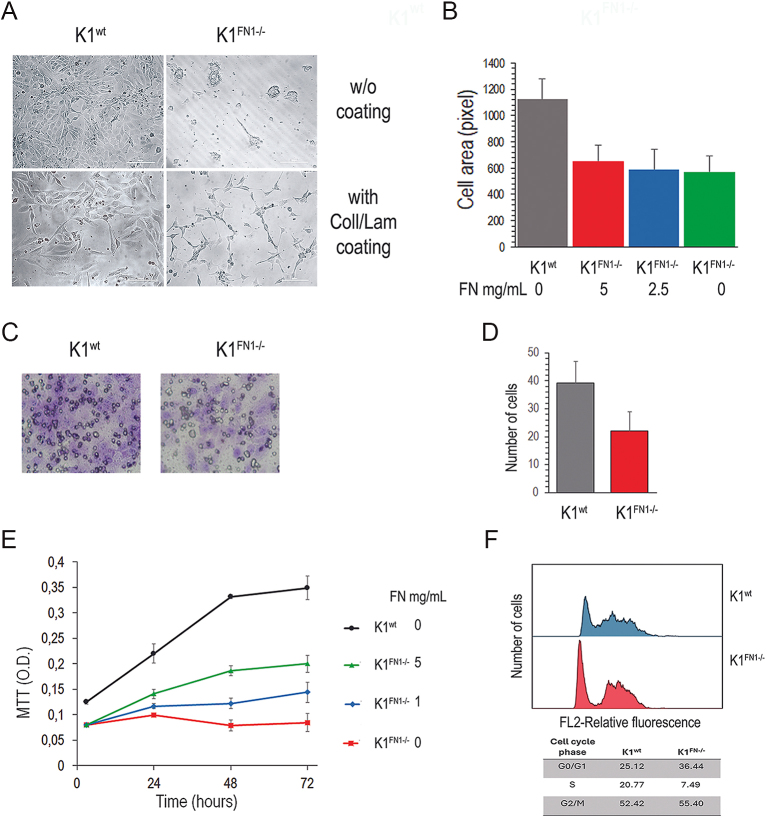
Migration and proliferation of FN1 knock-out K1 cell mutants. (A) K1^wt^ and K1^FN1−/−^ cells cultured for 4 days in serum-free medium in uncoated plates (top) or plates coated with collagen and laminin (bottom). In uncoated plates, K1^FN1−/−^ do not attach to the plate and form floating aggregates. In collagen/laminin coated plates, K1^FN1−/−^ adhere to the plate and spread, although less than K1^wt^. (B) Spreading of cells cultured for 72 h in collagen/laminin coated plates in the presence of soluble FN added to the serum-free medium. Cell spreading is not affected by soluble FN. (C) Representative images of K1^wt^ and K1^FN1−/−^ cells on the lower surface of Transwell inserts, photographed at 200× magnification. The lower chambers were filled with medium supplemented with 5% FBS, depleted of fibronectin. (D) Quantification of migrated cells to the lower surface of the Transwell inserts. Data represents the mean ± SD from three independent experiments. Statistical significance was determined using the Student’s *t*-test (*P* < 0.0001). (E) Cell proliferation measured as MTT absorbance in collagen/laminin coated plates in the presence of soluble FN in the serum-free medium. (F) Cell cycle analysis by flow cytometry after 48 h serum-free culture in collagen/laminin coated plates. DNA synthesis is reduced in K1^FN1−/−^ cells. *P < 0.0001.

K1 cells are more tumorigenic than BCPAP cells and produce more FN, making them the preferred choice for the knock-out experiments. The FN1 gene was knocked out using the CRISPR/Cas9 technique, resulting in K1 cell mutants (K1^FN1−/−^). FN expression was completely absent in K1^FN1−/−^ cells (Supplemental Fig. 1A and B (see section on Supplementary materials given at the end of the article)). K1^FN1−/−^ cells were propagated in medium containing 10% FBS as a source of FN. Under these conditions, K1^FN1−/−^ cells actively proliferated, although the proliferation rate was about 80% than the wild-type K1 cells (K1^wt^) (not shown). In the absence of serum, K1^FN1−/−^ cells did not adhere to the plate, remaining suspended and forming floating clusters over time (Fig. 5A). Coating the plates with collagen and laminin induced adhesion of K1^FN1−/−^ cells. When K1^wt^ and K1^FN1−/−^ cells were seeded onto collagen/laminin-coated plates and cultured in serum-free medium, both cell lines adhered to the plates, although the cell spreading of K1^FN1−/−^ was only 60.4% of that observed in K1^wt^ cells and the addition of soluble FN to the medium did not affect the spreading of K1^FN1−/−^ cells (Fig. 5B). A cell migration assay was conducted using Transwell plates, with the lower chamber filled with 5% FBS depleted of FN (Supplemental Fig. 1C). K1^FN1−/−^ cells displayed a 46.1% reduction of migration compared to K1 cells (Fig. 5C and D).

In the absence of FN, K1^FN1−/−^ cells showed minimal proliferation during the first 2 days (Fig. 5E), after which the cell number decreased. However, the proliferation of K1^FN1−/−^ cells was consistently stimulated by the addition of soluble FN to the medium. Without FN, DNA synthesis in K1^FN1−/−^ cells was significantly reduced (Fig. 5F).

### Inhibition of cell migration and proliferation by disintegrins

**Figure 6:**
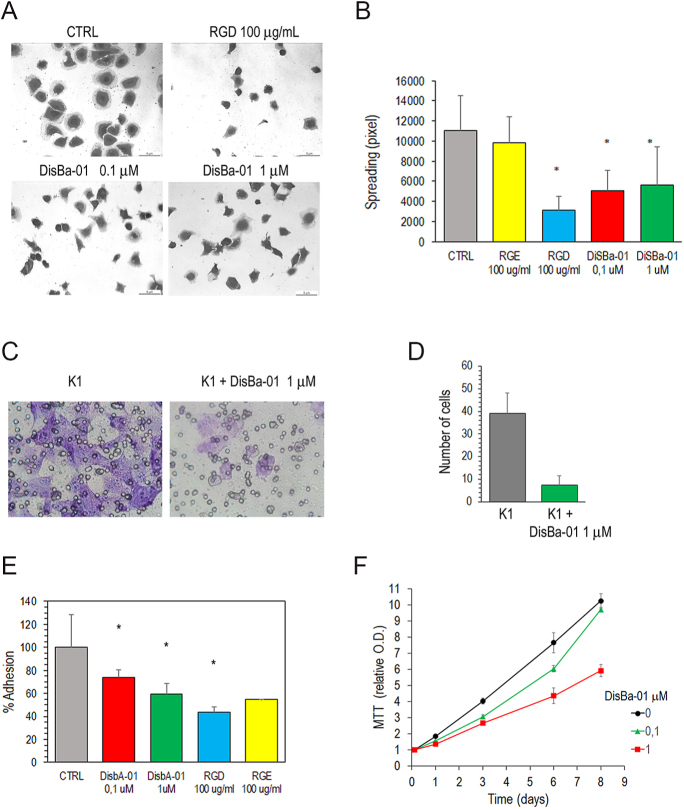
Effect of DisBa-01 on K1 cells. DisBa-01, RGD or RGE were added to K1 cells plated in medium with 10% FBS. (A) Cells observed after 3 h. (B) Surface area of the cells measured by the ImageJ Version 1.54i and expressed as the mean ± SD pixel. (C) Cell adhesion after 3 h treatment. (D) Representative images of K1 cells without or with 1 μM DisBa-01 on the lower surface of Transwell inserts, photographed at 200× magnification. The lower chambers were filled with medium supplemented with 5% FBS, depleted of fibronectin. (E) Quantification of migrated cells to the lower surface of the Transwell inserts. Data represent the mean ± SD from three independent experiments. Statistical significance was determined using the Student’s *t*-test (*P* < 0.0001). (F) Cell proliferation determined by MTT assay. Data are presented as relative to the 3 h time point. *P < 0.0001.

To test the FN/αvβ3 integrin binding as a novel target for the treatment of thyroid cancer, we tested DisBa-01, a recombinant His-tag fusion, RGD-disintegrin from *Bothrops alternatus* snake venom. This disintegrin binds to αvβ3 integrin with 100-fold higher affinity than α5β1 and acts as an antagonist of FN/αvβ3 binding (Montenegro *et al.* 2012). Although with a minor effect of RGD, DisBa-01 inhibited K1 cell spreading (72 and 54% reduction, respectively) (Fig. 6A and B). Accordingly, RGD induced a 56% inhibition of adhesion, while DisBa-01, a 46% inhibition (Fig. 6C). Migration was significantly inhibited by DisBa-01. In the Transwell assay, K1 cell migration toward the lower chamber containing FN-depleted 5% FBS was reduced to 18.7% (Fig. 6D and E). Cell proliferation also was inhibited by DisBa-01 in a dose-dependent fashion (6F). At 1 μM concentration, DisBa-01 achieved a 43% reduction by 6 days of culture.

## Discussion

Epithelial cells can transform into mesenchymal cells through a process known as epithelial–mesenchymal transition (EMT) (Hay 1995). During EMT, epithelial cells undergo cytoskeletal reorganization, lose their cell–cell adhesion, and acquire enhanced motility. Integrins implicated in the maintenance of epithelial polarity become downregulated, and integrins linked to increased cell proliferation, ECM interaction and cell migration are upregulated (Hamidi & Ivaska 2018). These changes are accompanied by alterations in signaling pathways, enabling the cells to degrade the ECM, migrate, and invade surrounding tissues. In thyroid cancer, alongside metabolic reprogramming, EMT is also characterized by alterations in integrin expression and modifications in the interactions between integrins and the ECM, particularly FN (Fedele *et al.* 2022), a key ECM glycoprotein that has been proposed to play an important role in cancer.

However, the role of FN in tumorigenesis and malignant progression remains highly controversial. While there is substantial evidence linking FN to advanced stages of cancer and poor prognosis when it is endogenously expressed in tumor cells, some studies suggest that FN expression in tumor cells can have a tumor-suppressive role, helping to maintain tissue architecture and prevent early tumor transformation and progression. This dual role highlights the complexity of FN function in cancer, depending on the tumor type, stage, and context of its expression (Lin *et al.* 2019). The interactions between PTC cells and the microenvironment, involving cell adhesion molecules and ECM components, have been a major focus of research, emphasizing the critical role of genetic alterations (Nucera *et al.* 2011).

The correlation between FN1 expression and clinicopathological features and prognosis in thyroid cancer was previously evaluated through the analysis of the TCGA dataset (Geng *et al.* 2021). However, the results were significantly underestimated because the analysis was conducted without considering the different histotypes and genetic mutations. The analysis of TCGA dataset in our study reveals that FN1 is highly expressed in BRAFV600E-positive PTC, where it shows an 83-fold increase. In contrast, PTC with RAS mutations exhibits the same FN1 expression levels as NT. In the absence of other mutations, such as in the TERT promoter, the BRAFV600E mutation confers only a modestly aggressive behavior to PTC and is a limited indicator of poor prognosis (OR = 1.8) (Xing *et al.* 2015). Compared to BRAFV600E, the contribution of FN1 to the aggressive behavior of PTC is considerably more significant, making it one of the most powerful prognostic indicators. The OR for recurrence in the higher quartiles of FN1 expression is markedly high (OR = 7.3).

This indicates that an elevated FN1 expression is associated with a higher risk of disease recurrence, highlighting its potential as a prognostic marker for unfavorable clinical progression. A correlation study would be valuable to determine whether the inclusion of FN1 expression analysis alongside conventional clinicopathological evaluation could improve the prognostication and management for PTC patients.

Integrins play a central role in cell motility, including the migration of cancer cells, by mediating interactions between the ECM and the cytoskeleton (Chastney *et al.* 2025). This function results from the cooperative action of multiple integrins. Junctional adhesion molecule (JAM)-A, a cell adhesion receptor localized at epithelial cell–cell contacts, forms a functional complex with α3β1 integrin to regulate collective cell migration of polarized epithelial cells (Tholmann *et al.* 2022). Through a distinct mechanism, integrin α5β1 functions as a key regulator of tumor cell migration and invasion by influencing cytoskeletal rearrangement, cell adhesion, and the production of matrix metalloproteinases (Hou *et al.* 2020). The role of the α3β1 integrin in cell motility and invasion was demonstrated in PTC cell lines (Mautone *et al.* 2021). In this context, the marked reduction in invasion observed in K1^FN1−/−^ cells, along with the inhibitory effect of DisBa-01 in the migration assay, highlights the crucial role of the FN/αvβ3 integrin interaction in regulating cell motility and invasion in this cell model.

In normal human thyroid cells, αvβ3/FN binding stimulates cell proliferation (Illario *et al.* 2005). Although adhesion to collagen and laminin supported appropriate spreading and morphology in K1^FN1−/−^ cells, the lack of self-produced FN resulted in impaired proliferation, an effect reversed upon the addition of soluble FN to the culture. ECM/integrin binding is also necessary for survival and 3 days of culture in suspension induce normal thyrocytes to anoikis (Vitale *et al.* 1998). However, unlike normal thyrocytes, where FN is essential for both survival and growth, FN is not required for the survival of tumoral K1 cells. FN1 knock-out K1 cell mutants cultured in suspension for 4 days did not proliferate but were also not induced to dead (Fig. 5C). In this condition, the number of FN1 knock-out K1 cells decreased only slightly during the first 2 days and then remained unchanged. This suggests that, while FN plays a significant role in growth and adhesion in both normal and tumoral thyroid cells, tumoral cells have developed adaptive mechanisms that enable survival in its absence. This adaptive mechanism may reduce the efficacy of a targeted therapy aimed at the FN1/integrin signaling pathway; however, it could still be effective as a cytostatic treatment.

The efficacy of various molecules with antagonistic activity against FN/integrin binding and anti-neoangiogenic properties has been investigated. Unfortunately, the peptide cilengitide and other small-RGD molecules with similar structures displayed a paradoxical effect, increasing angiogenesis at low concentrations. This effect is linked to conformational changes in αvβ3, which are associated with enhanced tumor growth *in vivo* (Stupp *et al.* 2014). In our cell model, DisBa-01, a recombinant His-tag fusion, RGD-disintegrin from *Bothrops alternatus* snake venom with antagonistic activity against the FN/αvβ3 binding, demonstrated a direct inhibitory effect on K1 cell proliferation and migration. We have previously demonstrated by surface plasmon resonance that DisBa-01 binds to αvβ3 integrin with high affinity, approximately 100-fold higher than its affinity for α5β1 integrin (Montenegro *et al.* 2017). This drug exerts multiple effects: it inhibits MMP-2 activation, induces autophagy, and disrupts VEGF/VEGFR2-mediated intracellular signaling, thereby inhibiting angiogenesis (Montenegro *et al.* 2012, Danilucci *et al.* 2019, Lino *et al.* 2019). Although DisBa-01 exhibits modest cytostatic effects in the thyroid cancer cell model studied, further *in vivo* evaluations of DisBa-01 and other disintegrins are warranted to assess their potential as therapeutic agents.

In conclusion, FN1 expression has significant prognostic value in PTC; the integrin–FN interaction plays a crucial biological role in this tumor and represents a potential target for targeted therapy.

## Supplementary materials



## Declaration of interest

The authors declare that there is no conflict of interest that could be perceived as prejudicing the impartiality of the work reported.

## Funding

This work did not receive any specific grant from funding agencies in the public, commercial, or not-for-profit sectors.
