# Supplementary figures and images for: Integrin–fibronectin interaction is a pivotal biological and clinical determinant in papillary thyroid carcinoma
Source: Endocr Relat Cancer. 2025 Jun 5;32(6):e250101. doi: 10.1530/ERC-25-0101 (PMC12150248; doi:10.1530/ERC-25-0101)

A

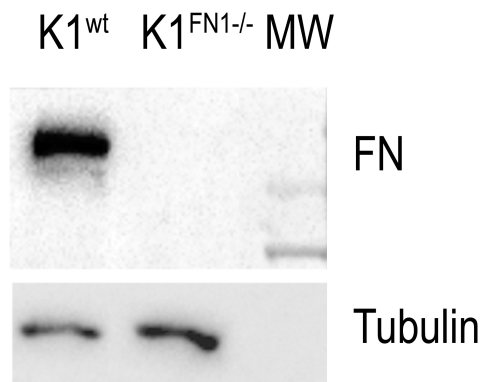

B

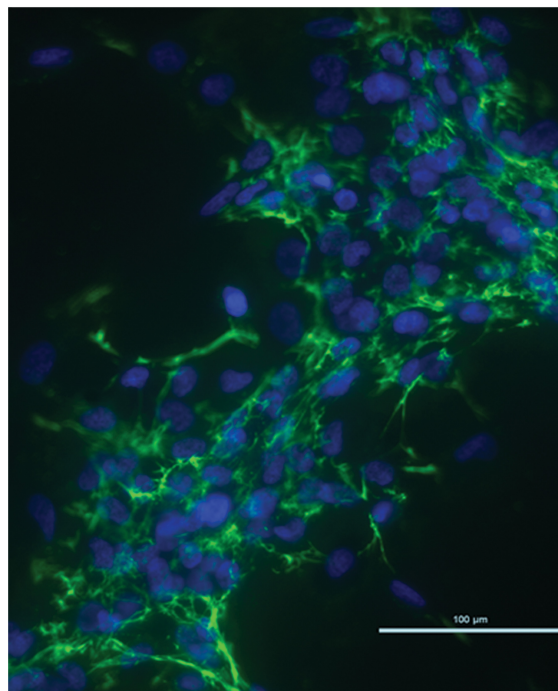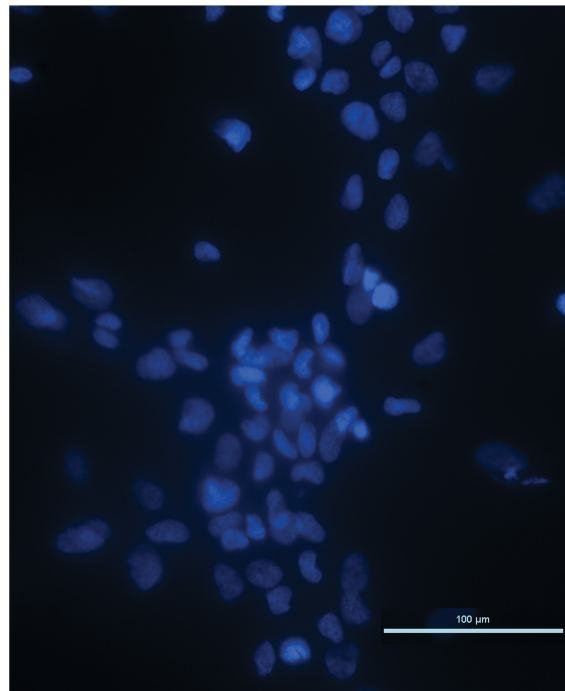

C

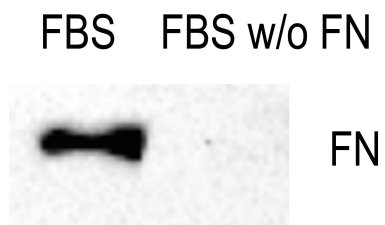

Supplement: Supplementary file 1 [file supplementary_materials.pdf]
